# Non-dispensed prescriptions – A nationwide descriptive study

**DOI:** 10.1016/j.rcsop.2024.100541

**Published:** 2024-11-16

**Authors:** Heini Kari, Fredriikka Nurminen, Hanna Rättö, Hanna Koskinen

**Affiliations:** a Research Unit, The Social Insurance Institution of Finland (Kela), Helsinki, Finland; b INVEST Research Centre, University of Turku, Turku, Finland

**Keywords:** Prescribing, Non-dispensed prescriptions, Medication adherence, Pharmacoepidemiology, Outpatient care

## Abstract

**Background:**

Medication non-adherence is associated with suboptimal health outcomes, higher mortality, and increased healthcare costs.

**Objective:**

The aim of this study was to estimate the number and share of non-dispensed prescriptions at a national level and in specific patient and medicine subgroups.

**Methods:**

The study was a nationwide retrospective register-based study. The data consisted of prescriptions prescribed in Finland in 2020 and dispensed between 2020 and 2022. A prescription was considered non-dispensed if it had not been dispensed within the two-year validity period. For each prescription, information on the patient's birth date, sex, and income as well as details of the prescribed medicine and physician's employment sector (public/private) were collected. Distributions and odds ratios (ORs) with corresponding 95 % confidence intervals (CI) were used in the analyses.

**Results:**

Of the 26 million prescriptions, 13.3 % were never filled. Over 1.7 million people (43.3 % of all people with prescriptions issued in 2020) had at least one non-dispensed prescription. The share of non-dispensed prescriptions was lower in men than women (12.9 % vs. 13.5 %; OR:0.95; CI:0.95–0.95). Compared to the youngest age group, the share of non-dispensed prescriptions was lower in the older age groups. The lowest share of non-dispensed medicines was in antineoplastic and immunomodulating agents (7.8 %) and in cardiovascular system medicines (8.1 %), whereas the highest was in dermatologicals (20.2 %). The proportion of non-dispensed prescriptions varied between medicine groups, from 5 % for thyroid therapy to 38 % for other nervous system drugs. The most frequently non-dispensed medications were paracetamol, ibuprofen, and salbutamol.

**Conclusion:**

The share and number of non-dispensed prescriptions varied across therapeutic areas, medicine groups, active pharmaceutical ingredients, and patient groups. Healthcare professionals should avoid unnecessary prescribing and improve medication adherence to ensure safer and more effective care.

## 1 Introduction

Prescribing medicines is the most important function used by physicians to treat illnesses, relieve symptoms, and prevent diseases.^1^ Rational prescribing is essential for safe and effective pharmacotherapy.^2^ It includes making a diagnosis, estimating prognosis, establishing the goals of pharmacotherapy, selecting the most appropriate treatment, and finally monitoring the effects of the treatment in a patient.^1^ Patients should be involved in the prescribing process, and their beliefs, expectations, and attitudes should contribute to the prescribing decisions. Additionally, patient involvement is essential in identifying previous clinical drug-related problems, which might affect the patient's adherence to selected pharmacotherapy.^3^

A large number of prescribed medicines are, however, never dispensed from pharmacies.^4^^,^^5^ The overall rates of non-dispensed prescriptions in different countries and studies have varied from 2.5 % in a study from Sweden to 12 % in Denmark and 22 % in the United States.^4^, ^5^, ^6^ In a study by Ekedahl & Månsson, the most common reason for not having a prescription filled was that the prescription was not needed.^7^ Furthermore, one quarter of the patients were not aware that an electronic prescription had been issued and sent to the pharmacy.

Medication non-adherence can be intentional or unintentional. Nevertheless, it is one of the major reasons why patients fail to reach their clinical goals, which often results in suboptimal health outcomes.^8^ Previous studies have shown that medication adherence to many treatments is poor, with estimates of 40 %–50 % of non-adherence to long-term therapies for chronic illnesses.^9^^,^^10^ Furthermore, medication non-adherence places a significant financial burden on healthcare systems^11^: it is estimated that medication non-adherence is associated with almost 200,000 premature deaths and costs Europe €80–€125 billion every year.^12^

Earlier, a majority of new therapies were prescribed during face-to-face appointments, but the COVID-19 pandemic accelerated the digital transformation also in the healthcare sector.^13^ In Finland, patients can nowadays easily request a prescription renewal electronically, and prescriptions can be renewed without the physician meeting the patient.^14^ In such case, the patient and the physician do not have an opportunity to discuss the pharmacotherapy, and empowering patients for medication use can only take place in a pharmacy, provided that the patient decides to pick up their medication.

Previous studies have shown that socio-demographic characteristics (e.g., age, sex, income), total number of medicines used, and higher co-payments may be associated with the non-dispensing of prescriptions.^15^, ^16^, ^17^, ^18^ However, the results of earlier studies have varied between countries, patient groups, and medicines, partly due to different reimbursement policies and healthcare system characteristics.^19^ Furthermore, due to a lack of comprehensive databases that could link prescribing and dispensing data at the national level, there is scarce knowledge of non-dispensed prescriptions that cover all prescriptions and dispensations, including prescriptions for both reimbursed and non-reimbursed medicines. Identifying which prescriptions remain unfilled and characteristics of patients who do not fill them can help to uncover barriers to medication adherence at a population level.

The aim of this study was to estimate the number of non-dispensed prescriptions at the national level as well as in specific patient and medicine subgroups.

## 2 Methods

The study was a nationwide retrospective register-based study.

### 2.1 Study setting

In Finland, all outpatient prescriptions are in electronic form. They are recorded in the national Prescription Centre, which is a centralised database of prescription and dispensation data in Kanta Services.^20^ Prescriptions can be issued by physicians employed by primary, secondary, or tertiary healthcare providers in both public and private sectors. Prescription medicines can be dispensed in any community pharmacy, as all pharmacies have access to the Prescription Centre.

All permanent residents in Finland are entitled to reimbursement for outpatient prescription medicines that are assessed as eligible for reimbursement by the Pharmaceuticals Pricing Board under the Ministry of Social Affairs and Health.^21^ The basic rate of reimbursement for all reimbursed medicines is 40 % of the retail price. In addition, there are two disease-based reimbursement categories for severe and chronic diseases covering either 100 % (fixed co-payment of €4.50 per purchase) or 65 % of the retail price.^22^ Furthermore, an annual co-payment ceiling on cumulative expenditure on reimbursed medicines (€592.16 in 2022) applies, after which patients will pay a fixed fee (€2.50 per product per dispensation) for the rest of the calendar year. For medicines not covered by the reimbursement system, patients pay the full cost themselves. In 2022, the National Health Insurance (NHI) scheme covered 81 % of all medicine dispensations in outpatient setting.^23^

### 2.2 Data and statistical analysis

The data were collected from the national Prescription Centre and the Dispensations reimbursable under the NHI scheme register. Prescription Centre includes all prescriptions prescribed for outpatient setting and their dispensations from community pharmacies, including both reimbursable and non-reimbursable prescriptions and dispensations, whereas the register of reimbursable dispensations includes information on reimbursements paid under the NHI scheme.^23^

The data consisted of prescriptions issued in 2020 and all dispensations between 2020 and 2022. In Finland, prescriptions are valid for a maximum of two years. Thus, prescriptions that had not been dispensed within the two-year validity period were considered as non-dispensed prescriptions. The study population included all people in Finland with at least one prescription issued for outpatient care in 2020.

For each prescription, information on the patient's birth date and sex were collected. The patient's age was defined as the age at the end of 2020. Details of the prescribed medicine and physician's employment sector (public/private) were also collected for each prescription. Furthermore, each prescription was linked to dispensations made based on said prescription. The total number of prescriptions per patient issued in 2020 was calculated to indicate the degree of polypharmacy. Excessive polypharmacy was defined as ≥10 prescriptions. Annual prescription medicine costs per person were defined as mean annual retail price costs for 2020–2022. Information on costs was collected from linked dispensations. When comparing the costs of dispensed and non-dispensed prescriptions, the costs for prescriptions with no dispensations were estimated based on dispensations in a same medicine group.

Information on yearly income per person in 2022 was based on the details from an electronic database of incomes information and linked to prescription data using pseudonymised personal identity codes.^24^ Income data include information on paid wages, pensions, and benefits. Patients were grouped into income classes based on their annual income in 2022. Patients with an income lower than 75 % of the median annual income were defined as having low income, patients with 75 %–200 % of the annual median income were defined as having middle income, and patients with an annual income higher than 200 % of the median income were defined as having high income. Income classification and income deciles were calculated only for individuals over 18 years old and only for individuals for whom we were able to link information on income.

Prescribed medicines were categorised according to the Anatomic Therapeutic Chemical (ATC) classification system.^25^ All medicines that were prescribed at least once during 2020 were collected. However, for the ATC therapeutic subgroup level and active pharmaceutical ingredient (API) reviews, a limit of 10,000 prescriptions was set in each medicine group or API for inclusion in the analysis. The limit was set to avoid random variations in therapeutic subgroups and APIs comprising few prescriptions.^5^ The share of reimbursed dispensations was estimated by comparing the number of reimbursed dispensations with the number of all dispensations between 2020 and 2022.

Distributions and odds ratios (ORs) with corresponding 95 % confidence intervals (CI) were used to analyse non-dispended prescriptions. Data processing and analysis were conducted using R statistical computing software (version 4.3.3).

### 2.3 Ethics statement

As the study was based on secondary register data, no ethics board approval was required according to Finnish legislation.^26^ The Social Insurance Institution of Finland (Kela) approved the use of the data for the current study. The data used in the study were fully pseudonymised before the authors accessed the data. All data preparation and linkage in the study were done with pseudo-identifiers, and the authors did not have access to information that could identify individual participants at any stage of the study. Legal restrictions prevent the open sharing of the pseudonymised data supporting the current study, as individual-level health data is considered highly sensitive and access is strictly regulated by law in Finland.^27^ Interested parties may, however, apply for permission to access the data from Findata and Statistics Finland.^28^

## 3 Results

Altogether, 26,010,962 prescriptions were prescribed to 3,975,114 patients in 2020. Fifty-eight per cent of the prescriptions were prescribed to women, and 68.6 % of all prescriptions were prescribed by a physician operating in the public sector (Table 1). The most prescribed medicine groups were medicines for nervous system (6.8 million prescriptions), cardiovascular medicines (3.3 million prescriptions), and medicines for musculoskeletal system (2.9 million prescriptions) (Table 2).

**Table 1:** Non-dispensed prescriptions in different subgroups.

Characteristics	All patients (n)	Unique patients that had at least one non-dispensed prescription (n)	Percentage of the patients that had at least one non-dispensed prescription (%)	Odds ratio [95 % confidence interval] (patients)	All issued prescriptions (n)	Non-dispensed prescriptions (n)	Percentage of the non-dispensed prescriptions (%)	Odds ratio [95 % confidence interval] (prescriptions)
Total	3,975,114	1,720,256	43.3	–	26,010,962	3,454,039	13.3	–

Sex								
Female	2,134,734	980,246	45.9	1	14,986,336	2,022,084	13.5	1
Male	1,811,677	725,007	40.0	0.79 (0.78–0.79)	10,786,376	1,395,272	12.9	0.95 (0.95–0.95)

Age (years)								
0–17	476,197	204,983	43.0	1	1,665,164	353,939	21.3	1
18–29	494,581	217,855	44.0	1.04 (1.03–1.05)	2,374,572	414,608	17.5	0.78 (0.78–0.79)
30–49	974,396	429,723	44.1	1.04 (1.04–1.05)	5,778,355	836,246	14.5	0.63 (0.62–0.63)
50–69	1,169,169	471,221	40.3	0.89 (0.89–0.90)	8,428,766	917,258	10.9	0.45 (0.45–0.45)
70+	860,676	396,415	46.1	1.13 (1.12–1.14)	7,762,648	931,441	12.0	0.51 (0.50–0.51)

Polypharmacy (prescriptions per year)								
1–2	1,220,769	244,021	20.0	1	1,783,383	279,741	15.7	1
3–7	1,587,802	659,347	41.5	2.84 (2.83–2.86)	7,339,425	1,017,521	13.9	0.87 (0.86–0.87)
8–10	444,796	266,406	59.9	6.00 (5.93–6.02)	3,950,906	513,280	13.0	0.80 (0.80–0.81)
11–15	380,113	266,933	70.2	9.44 (9.36–9.52)	4,815,066	613,810	12.7	0.79 (0.78–0.79)
>15	341,632	283,547	83.0	19.54 (19.35–19.73)	8,122,182	1,029,687	12.7	0.78 (0.78–0.78)

Total medicine costs (€ per year)^a^								
<20	388,750	130,515	33.6	1	731,558	200,693	27.4	1
20–99.99	1,003,989	360,186	35.9	1.11 (1.10–1.12)	3,270,549	614,816	18.8	0.61 (0.61–0.62)
100–299.99	1,074,526	43,460	40.3	1.34 (1.33–1.35)	5,921,615	825,759	13.9	0.43 (0.43–0.43)
300–599.99	572,650	269,995	47.1	1.77 (1.75–1.78)	4,834,599	567,939	11.7	0.35 (0.35–0.35)
600–999.99	337,591	172,079	51.0	2.06 (2.04–2.08)	3,534,784	389,615	11.0	0.33 (0.33–0.33)
1000+	553,957	310,391	56.0	2.52 (2.50–2.54)	7,580,182	768,673	10.1	0.30 (0.30–0.30)

Income 2022 (€ per year, 18+ years old)^b^								
<20,120, low income	1,029,590	450,539	43.8	1	8,496,755	928,883	10.9	1
20,120–53,652, middle income	1,595,452	688,153	43.1	0.97 (0.97–0.98)	10,846,599	1,343,485	12.4	1.15 (1.15–1.16)
53,652+, high income	413,251	168,646	40.8	0.89 (0.88–0.89)	2,417,282	308,668	12.8	1.19 (1.19–1.20)

Physician's employment sector								
Public	–	–	–	–	17,835,644	2,245,234	12.6	1
Private	–	–	–	–	8,168,522	1,207,826	14.8	1.20 (1.20–1.21)

Income was not available for 460,620 individuals of the 18+ years old study population.

a Mean annual prescription medicine costs 2020–2022.

b Inclusion criteria: persons at least 18 years old at the end of 2020.

**Table 2:** Non-dispensed prescriptions by different medicine classes (ATC main group level).

Main group of ATC	Percentage of non-dispensed prescriptions (%)	Number of non-dispensed prescriptions (n)	Total number of issued prescriptions (n)	Number of people with at least one non-dispensed prescription (n)
Gastrointestinal and metabolism (A)	16.9	441,289	2,613,138	332,741
Blood and blood-forming organs (B)	17.2	110,952	646,367	97,469
Cardiovascular system (C)	8.1	263,354	3,252,420	207,040
Dermatologicals (D)	20.2	236,222	1,168,479	194,767
Genitourinary system (G)	13.5	176,343	1,306,611	151,548
Systemic hormonal preparations (H)	9.2	58,621	634,207	52,104
Anti-infectives (J)	9.5	216,506	2,282,310	193,731
Antineoplastic and immunomodulating agents (L)	7.8	15,356	197,704	13,418
Musculoskeletal system (M)	14.0	416,473	2,974,711	346,749
Nervous system (N)	11.5	780,586	6,783,820	552,149
Antiparasitic (P)	11.2	16,413	146,673	15,340
Respiratory system (R)	14.9	368,375	2474,082	283,388
Sensory organs (S)	14.0	170,856	1,218,012	139,836
Various (V)	15.9	1252	7885	1106

A shown in Table 1, 13 % of all prescriptions (3.5 million prescriptions) were never filled during the maximum prescription validity period. Over 1.7 million people (43 %) had at least one prescription that was not filled, and 57 % of them were women. One fifth of the patients with a maximum of two prescriptions prescribed in 2020 had at least one non-dispensed prescription. Of those with excessive polypharmacy (more than 10 prescriptions per year), 76 % did not have at least one of their prescriptions filled. While the mean cost of all dispensations was €29.60 and the median cost was €14.30, the estimated mean cost for the non-dispensed prescriptions was €28.70 and the median cost was €16.10.

Men were less likely than women to have at least one non-dispensed prescription: 40.0 % of men and 45.9 % of women had at least one non-dispensed prescription (OR 0.79, 95 % CI 0.78–0.79) (Table 1). Compared to the youngest age group (0–17 years), those aged 50–69 were less likely to have at least one non-filled prescription (OR 0.89, 95 % CI 0.89–0.90). Patients in other examined age groups were significantly more likely to have at least one non-dispensed prescription than patients in the youngest age group. Compared to patients with 1–2 prescriptions, patients with 3 or more prescriptions were also significantly more likely to have at least one non-dispensed prescription during the study period. Furthermore, compared to patients with average annual medicine costs of less than €20.00, those with higher costs were more likely to have non-dispensed prescriptions. Compared to the lowest income category, those with middle income and high income were more likely to have at least one non-dispensed prescription (OR 0.97, 95 % CI 0.97–0.98 and OR 0.89, 95 % CI 0.88–0.89, respectively).

The share of the non-dispensed prescriptions of all prescriptions was lower with men than with women (12.9 % vs. 13.5 %; OR 0.95, CI 0.95–0.95) (Table 1). Compared to the patients in the youngest age group, the share of non-dispensed prescriptions was lower in older age groups, and compared to the patients with 1–2 prescriptions, the share of non-dispensed prescriptions was higher in patients with more prescriptions. Higher annual medicine costs were also associated with a lower share of non-dispensed prescriptions when compared to the patients with average annual medicine costs of less than €20.00. For those who had high yearly medicine costs (€1000+), the ratio of non-dispensed prescriptions was the lowest (10.1 %) with an OR of 0.30 [CI 0.30–0.30] compared to the lowest annual yearly medicine cost group. Patients belonging to the lowest income category (<€20,120 per year) had a lower rate of non-dispensed prescriptions (10.9 %) than those in the middle income (12.4 %; OR 1.15, 95 % CI 1.15–1.16) and high-income categories (12.8 %; OR 1.19, 95 % CI 1.19–1.20). Furthermore, 12.6 % of the prescriptions that were prescribed in the public sector and 14.8 % of the prescriptions issued in the private sector passed their expiry date without being dispensed (OR 1.20, 95 % CI 1.20–1.21).

Antineoplastic and immunomodulating agents (ATC main group L) and cardiovascular system medicines (ATC main group C) had the lowest share of non-dispensed medicines with 7.8 % and 8.1 %, respectively (Table 2). Furthermore, less than 10 % of systemic hormonal preparations (H) and anti-infectives for systemic use (J) remained non-dispensed. The highest share of non-dispensed prescriptions was in dermatologicals (D), 20.2 %, and in medicines that affect blood & blood forming organs (B), 17.2 %. The number of people that had at least one non-dispensed prescription was the highest in nervous system (N) medicines and in musculoskeletal system (M) medicines groups.

On the therapeutic subgroup level four groups had the highest rates of non-dispensed prescriptions (over 30 %): other nervous system drugs (N07) 38.1 %, drugs for constipation (A06) 37.6 %, vasoprotectives (C05) 37.4 %, and topical products for joint and muscular pain (M02) 33 % (Table 3). The top 20 list of non-dispensed prescriptions included multiple medicine groups that are typically not regularly taken. Furthermore, there were medicine groups with no or low reimbursement levels (e.g., vasoprotectives (C05), anesthetics (N01), vaccines (J07), and cough and cold preparations (R05)). On the contrary, the therapeutic subgroups that had the *lowest* non-dispensing rate include medicines for chronic and serious conditions, and a majority of the medicines in these subgroups are reimbursable in Finland. The percentage of non-dispensed prescriptions was lowest in the therapeutic groups of thyroid therapy (H03) 5.0 %, agents acting on the renin-angiotensin system (C09) 5.3 %, lipid modifying agents (C10) 6.2 %, calcium channel blockers (C08) 6.5 %, antigout preparations (M04) 7.2 %, and drugs used in diabetes (A10) 7.2 %.

**Table 3:** Top 20 therapeutic subgroups of medicines with the highest and lowest rates of non-dispensed prescriptions.

The highest rates of non-dispensed prescriptions by therapeutic subgroups of the medicines						The lowest rates of non-dispensed prescriptions by therapeutic subgroups of the medicines				
Therapeutic subgroup (ATC code)	Percentage of non-dispensed prescriptions (%)	Number of non-dispensed prescriptions (n)	Total number of issued prescriptions (n)	Share of reimbursed dispensations 2020–2022 (%)	Mean cost of the dispensation (€)	Therapeutic subgroup (ATC code)	Percentage of non-dispensed prescriptions (%)	Number of non-dispensed prescriptions (n)	Total number of issued prescriptions (n)	Mean cost of the dispensation (€)
Other nervous system drugs (N07)	38.1	37,116	97,493	89.0	70.21	Thyroid therapy (H03)	5.0	15,042	301,289	8.09
Drugs for constipation (A06)	37.6	105,444	280,416	65.8	28.31	Agents acting on the renin-angiotensin system (C09)	5.3	45,029	851,496	13.65
Vasoprotectives (C05)	37.4	29,606	79,254	0.0	22.48	Lipid modifying agents (C10)	6.2	40,412	655,892	11.00
Topical products for joint and muscular pain (M02)	33.0	51,453	155,999	36.3	25.77	Calcium channel blockers (C08)	6.5	25,214	387,992	10.17
Other gynecologicals (G02)	29.8	16,420	55,091	15.7	79.44	Antigout preparations (M04)	7.2	4060	56,515	10.45
Antihemorrhagics (B02)	29.2	8382	28,681	99.9	1330.98	Drugs used in diabetes (A10)	7.2	44,854	621,707	82.91
Antifungals for dermatological use (D01)	28.5	45,040	157,959	72.5	22.47	Beta blocking agents (C07)	7.5	50,056	670,722	9.81
Vitamins (A11)	27.1	19,237	71,007	4.4	8.12	Antineoplastic agents (L01)	7.5	2181	29,033	1374.04
Anesthetics (N01)	26.3	5339	20,319	0.3	33.38	Immunosuppressants (L04)	7.7	8151	105,272	620.33
Antianemic preparations (B03)	25.2	36,031	142,889	14.0	43.57	Endocrine therapy (L02)	7.9	3963	50,306	470.64
Antidiarrheals, intestinal anti-inflammatory/anti-infective agents (A07)	23.6	41,018	174,203	70.8	77.27	Antibacterials for systemic use (J01)	8.0	149,836	1,875,473	16.92
Other dermatological preparations (D11)	22.9	15,662	68,348	16.3	113.97	Immunostimulants (L03)	8.1	1061	13,093	782.31
Antiemetics and antinauseants (A04)	21.9	8750	39,975	84.1	63.87	Psycholeptics (N05)	8.1	171,572	2,116,051	21.35
Drugs for functional gastrointestinal disorders (A03)	21.3	37,000	173,823	26.4	16.04	Antihypertensives (C02)	8.4	1887	22,600	102.27
Stomatological preparations (A01)	21.1	14,963	70,805	0.0	14.44	Antiepileptics (N03)	8.4	36,850	438,331	42.87
Vaccines (J07)	19.7	43,663	221,729	0.0	61.91	Antiprotozoals (P01)	8.5	9785	114,805	22.57
Cough and cold preparations (R05)	19.5	33,558	172,077	0.1	20.42	Anti-parkinson drugs (N04)	8.9	6971	78,045	60.49
Corticosteroids, dermatological preparations (D07)	18.9	119,119	630,975	85.1	18.30	Urologicals (G04)	9.4	41,794	447,198	26.14
Mineral supplements (A12)	18.6	44,264	238,528	61.0	11.38	Drugs for treatment of bone diseases (M05)	9.6	3772	39,146	128.19
Antibiotics and chemotherapeutics for dermatological use (D06)	18.5	27,700	149,876	24.6	23.85	Psychoanaleptics (N06)	9.8	138,408	1,417,927	21.43

On the API level, there were seven medicines for which more than half of the prescriptions were not filled (Table 4). These include prednisolone products for haemorrhoids (C05AA04) 63.3 %, sodium picosulfate (A06AB08) 59.6 %, alginic acid (A02BX13) 54.0 %, macrogol (A06AD15) 52.9 %, ketoconazole (D01AC08) 51.2 %, miconazole (D01AC02) 51.1 %, and varenicline (N07BA03) 51.0 %. The majority of the medicines included in the top 20 list of the highest rates of non-dispensed prescriptions are also available as over-the-counter medicines and 17 of them are not reimbursable. In terms of the total number of non-dispensed prescriptions, the top five APIs were paracetamol (N02BE01), ibuprofen (M01AE01), salbutamol (R03AC02), pantoprazole (A02BC02), and etoricoxib (M01AH05) (Table 5).

**Table 4:** Top 20 active pharmaceutical ingredients (APIs) with the highest and lowest rates of non-dispensed prescriptions.

The highest rates of non-dispensed prescriptions by active pharmaceutical ingredients							The lowest rates of non-dispensed prescriptions by active pharmaceutical ingredients^c^				
Active pharmaceutical ingredient (ATC code)	Percentage of non-dispensed prescriptions (%)	Number of non-dispensed prescriptions (n)	Total number of issued prescriptions (n)	Share of reimbursed dispensations 2020–2022 (%)	Mean cost of the dispensation (€)	Available as an over-the-counter medicine (OTC), non-reimbursable (NR)	Active pharmaceutical ingredient (ATC code)	Percentage of the non-dispensed prescriptions (%)	Number of non-dispensed prescriptions (n)	Total number of issued prescriptions (n)	Mean cost of the dispensation (€)
Prednisolone (C05AA04)	63.3	9610	15,185	0.0	22.61	OTC, NR	Bisoprolol and thiazides (C07BB07)	3.4	346	10,184	23.45
Sodium picosulfate (A06AB08)	59.6	10,094	16,930	0.0	16.88	OTC, NR	Telmisartan and diuretics (C09DA07)	3.4	566	16,508	17.32
Alginic acid (A02BX13)	54.0	12,660	23,467	0.0	22.98	OTC, NR	Enalapril and diuretics (C09BA02)	3.5	745	21,432	15.52
Macrogol (A06AD15)	52.9	49,029	92,627	0.0	31.10	OTC, NR	Clozapine (N05AH02)	3.6	4225	117,670	30.67
Ketoconazole (D01AC08)	51.2	6420	12,536	0.0	25.77	OTC, NR	Alprazolam (N05BA12)	3.7	1882	50,659	14.39
Miconazole (D01AC02)	51.1	8393	16,436	0.0	16.41	OTC, NR	Valsartan and amlodipine (C09DB01)	3.8	480	12,667	22.94
Varenicline (N07BA03)	51.0	29,285	57,399	93.0	141.40		Valsartan and diuretics (C09DA03)	3.9	729	18,489	19.49
Retinol (S01XA02)	49.5	5293	10,692	0.0	21.59	OTC, NR	Losartan and diuretics (C09DA01)	4.0	2138	53,745	19.48
Estriol (G03CA04)	48.0	12,265	25,557	4.7	29.13	OTC^a^, NR^b^	Candesartan and diuretics (C09DA06)	4.1	1392	33,845	29.49
Acetylsalicylic acid (B01AC06)	46.3	30,504	65,912	0.0	3.11	OTC, NR	Flecainide (C01BC04)	4.1	418	10,098	36.47
Terbinafine (D01AE15)	45.2	8822	19,528	38.2	13.40	OTC^a^, NR^b^	Metformin and sitagliptin (A10BD07)	4.2	733	17,316	100.42
Influenza, vaccine (J07BB02)	42.5	7012	16,515	0.0	18.64	NR	Zolpidem (N05CF02)	4.3	5851	136,575	6.89
Xylometazoline (R01AA07)	41.6	5505	13,219	0.0	8.58	OTC, NR	Dapagliflozin (A10BK01)	4.4	893	20,322	108.67
Ferrous glycine sulfate (B03AA01)	39.6	17,822	44,997	0.0	7.75	OTC, NR	Empagliflozin (A10BK03)	4.7	2227	47,437	106.95
Ferric oxide polymaltose complexes (B03AB05)	39.0	4298	11,024	0.0	31.38	OTC, NR	Zopiclone (N05CF01)	4.8	14,877	311,733	7.69
Saccharomyces boulardii (A07FA02)	39.0	20,555	52,735	0.0	20.25	OTC, NR	Brinzolamide (S01EC04)	4.9	682	14,044	21.59
Opium derivatives and expectorants (R05FA02)	38.9	6750	17,366	0.0	23.74	OTC^a^, NR	Simvastatin (C10AA01)	4.9	7441	152,639	9.29
Diclofenac (M02AA15)	37.0	38,088	103,007	0.0	31.53	OTC^a^, NR	Levothyroxine sodium (H03AA01)	4.9	14,046	287,845	7.55
Magnesium hydroxide (G04BX01)	36.6	5709	15,605	0.0	4.78	OTC, NR	Tamsulosin and dutasteride (G04CA52)	5.0	1687	33,599	23.83
Colecalciferol (A11CC05)	35.0	12,269	35,053	0.0	4.79	OTC^a^, NR	Ezetimibe (C10AX09)	5.2	2474	47,731	9.91

a Some of the medicines included are available only by prescription.

b Group includes some medicines that are reimbursable.

c All the APIs are reimbursable.

**Table 5:** The most frequently non-dispensed prescriptions (active pharmaceutical ingredients, APIs).

Active pharmaceutical ingredient (ATC code)	Non-dispensed prescriptions (n)	Total number of issued prescriptions (n)	Percentage of non-dispensed prescriptions (%)	Number of people who had at least one non-dispensed prescription (n)	Prescriptions by public sector physicians (%)	Share of reimbursed dispensations 2020–2022 (%)	Mean cost of the dispensation (€)
Paracetamol (N02BE01)	248,074	1,362,151	18.2	229,792	73.8	92.1	8.59
Ibuprofen (M01AE01)	155,813	1,18,977	13.1	146,329	63.3	94.0	13.00
Salbutamol (R03AC02)	69,308	442,666	15.7	66,058	65.8	97.0	12.35
Pantoprazole (A02BC02)	67,872	550,118	12.3	64,506	71.4	100.0	7.01
Etoricoxib (M01AH05)	56,369	458,833	12.3	52,391	38.3	99.5	9.81
Macrogol (A06AD15)	49,029	92,627	52.9	46,680	88.9	0.0	31.10
Codeine and paracetamol (N02AJ06)	48,546	461,695	10.5	46,484	62.6	88.7	9.82
Pseudoephedrine, combinations (R01BA52)	43,218	220,885	19.6	41,391	31.5	0.0	31.46
Artificial tears and other indifferent preparations (S01XA20)	42,591	202,000	21.1	38,143	42.4	98.3	32.11
Cefalexin (J01DB01)	41,819	489,805	8.5	39,867	55.4	93.4	16.50
Diclofenac (M02AA15)	38,088	103,007	37.0	37,187	57.0	0.0	31.53
Naproxen (M01AE02)	36,083	219,955	16.4	34,792	50.5	77.7	11.87
Chloramphenicol (S01AA01)	34,739	293,600	11.8	30,295	56.3	0.0	15.71
Melatonin (N05CH01)	34,160	255,335	13.4	32,780	68.3	0.0	14.38
Tizanidine (M03BX02)	32,346	285,716	11.3	31,108	39.1	99.9	12.57
Acetylsalicylic acid (B01AC06)	30,504	65,912	46.3	29,326	87.7	0.0	3.11
Varenicline (N07BA03)	29,285	57,399	51.0	18,648	56.4	93.0	141.40
Estradiol (G03CA03)	29,194	255,134	11.4	26,824	34.2	82.3	19.82
Prednisolone (H02AB06)	28,956	246,227	11.8	26,265	76.3	71.2	7.19
Cetirizine (R06AE07)	27,945	178,295	15.7	27,132	73.9	94.3	9.49

The lowest rates of non-dispensed prescriptions were for APIs used to manage chronic conditions, such as hypertension, schizophrenia, and type 2 diabetes (Table 4). Medicines that had the lowest rates of non-dispensed prescriptions were bisoprolol and thiazides (C07BB07) 3.4 %, telmisartan and diuretics (C09DA07) 3.4 %, enalapril and diuretics (C09BA02) 3.5 %, clozapine (N05AH02) 3.6 %, and alprazolam (N05BA12) 3.7 %. Interestingly, eight out of twenty medicines with the lowest non-dispensation rates were combination medicines containing two active ingredients.

## 4 Discussion

Thirteen per cent of all prescriptions prescribed in Finland in 2020 passed their expiry date without being dispensed. We found that excessive polypharmacy (≥10 medicines) and older age were associated with having at least one non-dispensed prescription. The results on medicine subgroups align with other studies on non-dispensation and non-adherence: the share and number of non-dispensed prescriptions varied, for example, across therapeutic areas, medicine groups, and APIs.^15^^,^^17^^,^^29^

Here, the overall rate of non-dispensed prescriptions is similar to the results of a Norwegian study, where 12 % of electronic prescriptions were never dispensed.^5^ In a Swedish study, however, the rate was lower at only 2.5 %,^6^ whereas a study from the United States found that 22 % of all e-prescriptions were never filled.^4^ In some previous studies, the focus has been on primary medication non-adherence,^15^^,^^18^^,^^19^^,^^30^^,^^31^ which means that the patient does not redeem their first prescription for a new medication.^32^ For example, a meta-analysis by Cheen et al. showed that one in six patients with chronic diseases are nonadherent to new medications.^15^ Furthermore, in a Danish study, the overall rate of primary non-adherence was 9.3 %,^18^ and a study from Spain found a 17.6 % prevalence.^30^

In Finland, medicines for chronic and serious conditions are usually reimbursed, and the costs for the patient are rarely high. In our study, the lowest share of non-dispensed prescriptions was in antineoplastic and immunomodulating agents (ATC group L) and in cardiovascular system medicines (C), whereas the highest share was in dermatologicals (D). The proportion of non-dispensed prescriptions varied between the APIs from 3.4 % for bisoprolol and thiazides to 63 % for prednisolone products for haemorrhoids. The most frequently non-dispensed prescriptions were for paracetamol, ibuprofen, and salbutamol, which is a rather similar result compared to the Norwegian top 3, which included paracetamol, salbutamol, and diclofenac.^5^ Interestingly, nine of the twenty APIs in the list of lowest percentages of non-dispensed prescriptions were single-pill combination (SPC) therapy treatments. In previous studies, it has been shown that SPC therapy in hypertension leads to improved medication adherence and persistence compared to free-equivalent combination therapy, which may lead to better blood pressure control in patients with hypertension.^33^

In Finland, a majority of the top 20 most non-dispensed medicines are also available without a prescription as OTC medicines and not included in the reimbursement system (e.g., low dose acetylsalicylic acid, macrogol, xylometazoline). Because of the dispensing fee of €2.39, the patient pays more if they want to fill their prescription instead of buying the medicine as an OTC medicine. Many of these medicines are important for the patients, and it is not known whether the adherence is lower among the group of patients purchasing their medications without prescriptions.

During the study period, medicine shortages were common in Finland,^34^ which might have affected the prescribing and dispensing rates of some APIs. Furthermore, physicians might have prescribed extra prescriptions for several medications for the same diagnosis to ensure that the patient is able to continue their pharmacotherapy despite the ongoing shortage. However, for some APIs, such as varenicline used for smoking cessation, there were no equivalent substitute medicines available.

Previous studies have suggested several reasons associated with the non-dispensing of prescriptions. These include, for example, socio-demographic characteristics, number of medicines, and higher co-payments.^15^ In our study, compared to the patients in the youngest age group, the share of non-dispensed prescriptions was lower in older age groups. We also found that patients with more prescriptions were significantly more likely to have at least one non-dispensed prescription compared to patients with 1–2 prescriptions. Furthermore, compared to patients with the smallest average annual medicine costs, those with higher costs were more likely to have non-dispensed prescriptions. Some of the non-dispensed prescriptions might be prescriptions that the patient was not even aware of, particularly if the patient had numerous prescriptions or they did not visit the pharmacy regularly. In a study by Ekedahl & Månsson,^7^ the most common reported reason behind non-redemption was that the prescription was not needed. Unintentional non-compliance was also common and reported by 28 % of patients, most of whom were not aware that a prescription had been transmitted to the pharmacy.

We did not find major differences in the proportions of non-dispensed prescriptions between the three income classes. However, in our study, the lower income class was associated with a lower non-dispensing rate. Conversely, in a Danish study, the higher income class was found to be significantly associated with lower rates of non-dispensed prescriptions.^18^ Nevertheless, while the purpose of the study by Pottegård et al. was to study the extent to which patients fail to have the first prescription for a new medicine filled, our study included all the prescriptions included in the nationwide database. Furthermore, we used personal annual income, whereas family income was utilised in the Danish study. Utilised medicines might differ between income classes, which should be further studied.

The comprehensive national coverage and high overall quality of the used prescription and dispensation data is the strength of our study. In addition, our study included the whole validity period of the prescriptions. While in many previous studies the data have been limited, for example, to some disease or medicine groups,^15^^,^^35^ or to a certain area of a country,^6^^,^^7^ our study includes all prescriptions and dispensations as well as all medicine groups and APIs.

Despite its strengths, the study also has some limitations. As our study included all prescriptions, some overlapping prescriptions for the same medicines and some patients might have been included. Obviously in such case, the patient does not, in general, have the duplicate prescription filled. For patient income, we were only able to match patient annual income data for 86.3 % of those over 18 years old. Furthermore, the dataset for income information does not include information on, for example, capital income, self-employment income, or income support. It should also be noted that 2020 was the first year of the COVID-19 pandemic. Barriers in accessing healthcare and changes in the availability of services as well as other changes brought about by the exceptional global situation during our study period might be reflected in our results.^36^ For example, in some medicine groups, there was a statistically significant reduction in the level of monthly and yearly number of outpatient prescriptions (e.g., antibiotics).^37^ However, for regularly used medications, no significant changes in yearly utilisation trends were observed, for example for antidiabetic medicines.^38^ As we observe the prescriptions prescribed in 2020 for the entire duration of their validity (up to 2 years), the exceptional situation in 2020 is unlikely to bias our findings on the non-dispensing of issued prescriptions.

The low proportion of non-dispensed medications in some medicine groups might indicate a high-level of medication adherence. However, it should be taken into account that prescribed and dispensed medicines are not necessarily taken. With these data, we are not able to identify the secondary non-adherence, i.e., situations where the patient intentionally or unintentionally does not use the medicine dispensed as prescribed. We are only certain that the patients redeemed or did not redeem their medications. However, particularly in chronic conditions, linking prescribing and dispensing records can serve as a proxy for patients' adherence to medicines.^39^ Healthcare professionals should try to identify both primary and secondary non-adherence, and they should improve medication adherence as it is a global problem that jeopardises health and economic outcomes for individuals and society.^40^ Necessary medicines should be prescribed, dispensed, and taken, but for ensuring medication safety and avoiding extra medication costs, unnecessary prescribing of prescriptions for patients should be avoided. Targeting public health interventions, such as patient education, financial assistance, and support for medication adherence, to the most relevant patient groups is essential for maximizing their effectiveness and improving health outcomes. Furthermore, clinicians should ensure that their prescribing practices are rational, and that the medications they prescribe are necessary and accessible to patients.

## Funding

The authors received no specific funding for this work.

## CRediT authorship contribution statement

**Heini Kari:** Writing – review & editing, Writing – original draft, Project administration, Methodology, Conceptualization. **Fredriikka Nurminen:** Writing – review & editing, Visualization, Methodology, Formal analysis, Data curation, Conceptualization. **Hanna Rättö:** Writing – review & editing, Methodology, Conceptualization. **Hanna Koskinen:** Writing – review & editing, Methodology, Conceptualization.

## Declaration of competing interest

The authors declare that they have no competing interests.

## Data Availability

Due to legal restrictions and data protection regulations of the administrative sources providing individual-level register data, the authors do not have the permission to make sensitive personal data available. Interested parties may apply for permissions to access the data from the centralised data permit authority Findata (https://www.findata.fi/en/), info@findata.fi.
